# An uncommon complication of a minimally invasive procedure

**DOI:** 10.1007/s12055-025-01962-3

**Published:** 2025-05-15

**Authors:** Valerio Massimo Magro, Andrea Sorbino, Nicola Manocchio, Gianluca Massaro, Gaetano Chiricolo, Concetta Ljoka, Calogero Foti

**Affiliations:** 1 Department of Internal Medicine and Geriatry, Echocardiography Unit, University of Campania “Luigi Vanvitelli”, Piazza L. Miraglia 2, 80100 Naples, Italy; 2 Physical and Rehabilitation Medicine, Department of Clinical Sciences and Translational Medicine, Tor Vergata University, 00133 Rome, Italy; 3 Department of Cardiology, Tor Vergata University Hospital, 00133 Rome, Italy; 4 Department of Biomedicine and Prevention, University of Rome Tor Vergata, 00133 Rome, Italy

**Keywords:** Decision-making, Aortic stenosis, TAVI, Heart Aneurysm

## Abstract

Decision-making for intervention in symptomatic aortic stenosis should balance the risks of surgery and of transcatheter aortic valve implantation (TAVI). TAVI is a well-established technique for treating elderly and high-risk patients with aortic stenosis using a variety of different surgical approaches (a retrograde transfemoral, transaxillary, transaortic or an antegrade transapical approach). The transapical approach requires the involvement of the heart surgeon and is now limited to cases where the procedure cannot be performed by alternative approaches. We report the case of an 87-year-old patient with severe peripheral arterial disease and a history of TAVI via transapical approach, who presented with a syncopal episode 4 years post-procedure. Imaging revealed a left ventricular apex aneurysm, likely related to the previous transapical TAVI. This case underscores the importance of thorough pre-procedural assessment and long-term follow-up in patients undergoing alternative TAVI access routes, as well as the need for heightened awareness of rare but significant complications such as ventricular pseudoaneurysm. Careful patient selection and individualized procedural planning remain essential to optimize outcomes in this complex population.

Decision-making for intervention in symptomatic aortic stenosis should balance the risks of surgery and of transcatheter aortic valve implantation (TAVI). The latter is a well-established technique for treating elderly and high-risk patients with aortic stenosis using a variety of different surgical approaches (a retrograde transfemoral, transaxillary, transaortic or an antegrade transapical approach). The transapical approach requires the involvement of the heart surgeon and is now limited to cases where the procedure cannot be performed by alternative approaches [1]. We studied an 87-year-old patient suffering from peripheral arterial disease involving the carotids, and also the femoral arteries, who came to the emergency department for a syncopal episode. The patient had a history of severe aortic stenosis and had undergone TAVI 4 years earlier. The patient had already undergone coronary angiography the year before the TAVI implantation, for a non-ST-elevation myocardial infarction (NSTEMI), which had shown a stenosis in the right coronary artery and was treated with percutaneous coronary intervention (PCI) with a drug-eluting stent (DES). In preparation for the TAVI procedure, a new coronary angiography was performed. The examination revealed critical in-stent restenosis of the right coronary artery. The common trunk was free of lesions, while the anterior descending artery showed diffuse wall infiltrates but no calcific stenosis. Additionally, the circumflex artery was free of any critical lesions. For this reason, a balloon angioplasty (plain old balloon angioplasty (POBA)) was performed on the right coronary artery. Due to the presence of severe arterial disease, a trans-apical approach with left mini-thoracotomy was performed, with implantation of an aortic bioprosthesis with a transcatheter cardiac valve Edwards Sapien 3 Ultra n. 26 mm. During the 4 years, the patient remained eupnoeic on room air for the duration of the follow-up with occasional mild dyspnoea reported (grade 1 according to the Medical Research Council). An echocardiogram was performed which showed the presence of a rounded image, with hyperechogenic borders and anechoic content, at the apical level of the left ventricle (Fig. 1). We decided to perform a computed tomography scan, which confirmed the presence of a round-shaped structure in the pericardial area at the apical level of the left ventricle, likely attributable to the outcomes of the TAVI procedure (Fig. 2). An even more suggestive image was visualized by the hybrid technique with positron emission tomography-computed tomography (digital PET-CT) with fludeoxyglucose F18 (18F-FDG) (219 MBq e.v.), in which the presence of the collection localized near the cardiac apex was observed, characterized by ring-shaped uptake (standardized uptake value (SUV)—max 15.7) (Fig. 3). Based on these findings, we diagnosed a cardiac apex aneurysm as a result of TAVI procedure with transapical antegrade access. Transapical access for TAVI is one of the alternative access routes, preferred when the transfemoral or transaxillary route is not feasible, as in our patient [2]. This approach is not free of risks and complications that may affect the outcome of the treatment of aortic valve disease. For example, in a series of over 400 cases, of which only 127 were transapical, Lange et al. demonstrated the existence of complications, even significant ones requiring subsequent interventions, mostly involving the aorta [3]. Similarly, cases of coronary occlusion have been described [4]. A large study comparing survival of patients undergoing TAVI with different approaches showed a lower survival of patients treated with the transapical approach compared to the subclavian approach [5]. To our knowledge, pseudoaneurysms of the left ventricle have been reported infrequently in the medical literature. However, a few cases have been described after the transapical approach, often in elderly patients over 80 years of age [6–8]. Although the strict causal relationship is a matter of discussion and there is an open debate whether this finding should be considered a complication of TAVI or not, the clinical case examined highlights the need to study these patients very carefully, also to better define not only the type of surgical approach best suited to them, but also the probability of incurring this type of problem.

**Fig. 1:**
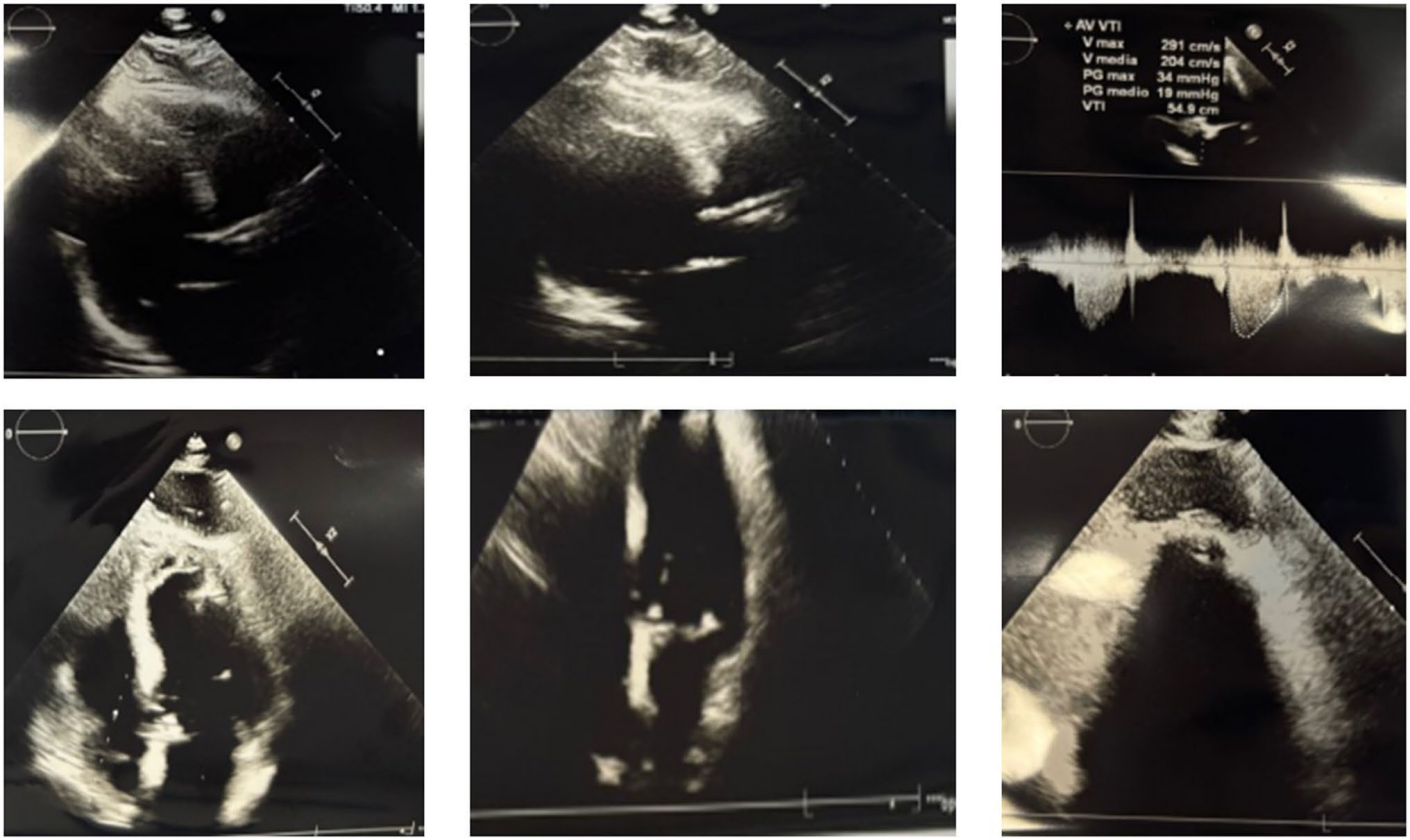
Echocardiogram of the patient who underwent a trans-apical antegrade TAVI procedure for aortic stenosis approximately 4 years ago. Images in different acoustic windows. The echocardiogram showed a slightly enlarged, hypertrophic left ventricle with normal contractility, together with the presence of a rounded image, with hyperechogenic margins and anechoic content, at the apical level of the left ventricle. The trans-prosthetic gradient was also measured at the TAVI level, which was found to be 19 mm Hg

**Fig. 2:**
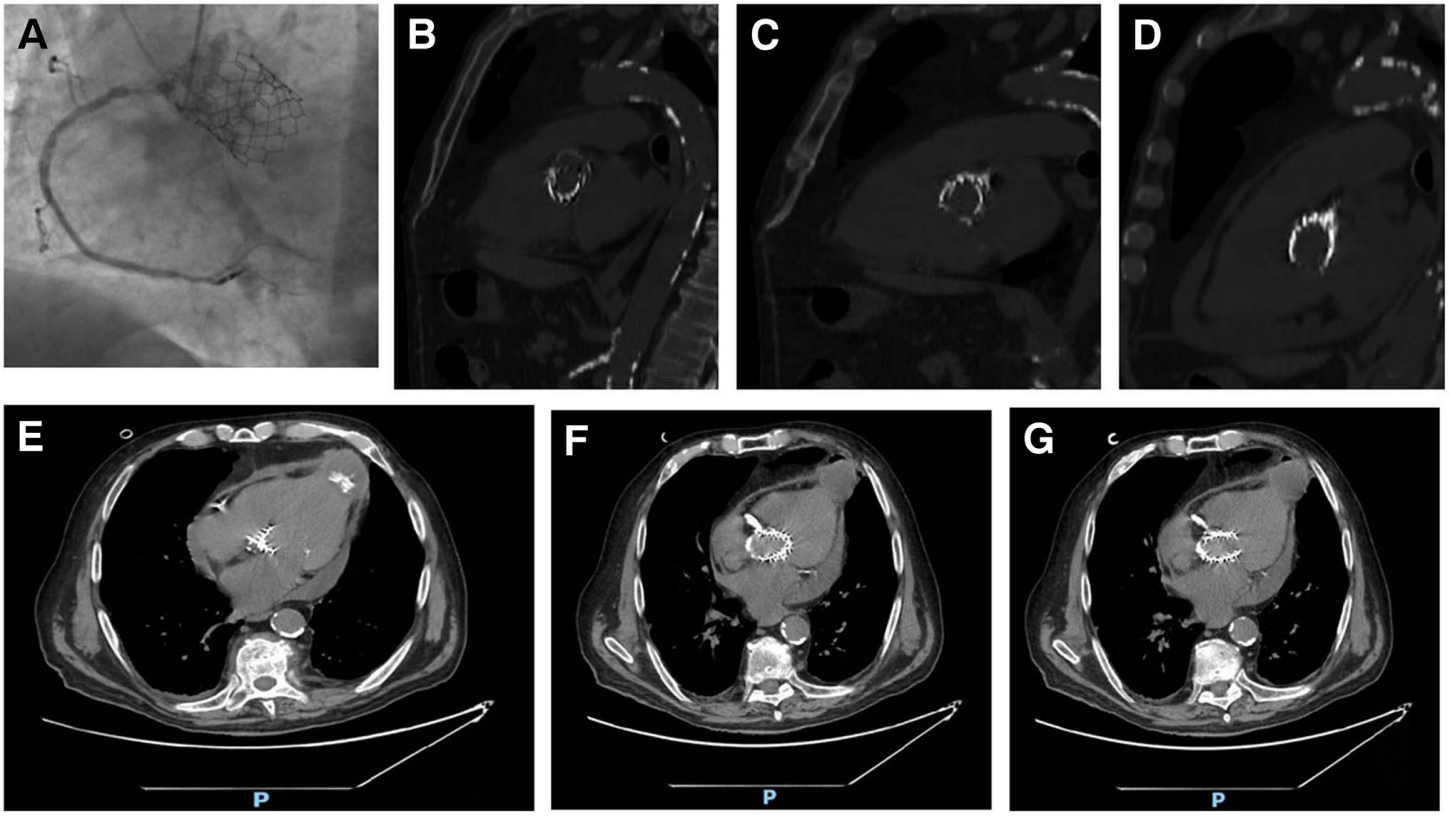
Coronary angiography and computed tomography images. Coronary angiography (**A**) shows the TAVI with the prosthesis correctly positioned. A computed tomography performed a few months after admission (**B**–**D**) for TAVI documented in the CT scans in the intrapericardial area, at the cardiac apex, the appearance of a 40 × 33 mm formation with partially calcified walls, the presence of fibrocalcified atheromatous aorta included in the acquisition volume, severe coronary calcifications, and a right coronary stent. The results of aortic valve replacement were confirmed. The CT scan performed during our admission (**E**–**G**) confirmed the presence of a “roundish image in the pericardial area at the apical level of the left ventricle to be referred to probable results of TAVI.” Based on these findings, we diagnosed a cardiac apex aneurysm as a result of TAVI intervention with trans-apical antegrade access

**Fig. 3:**
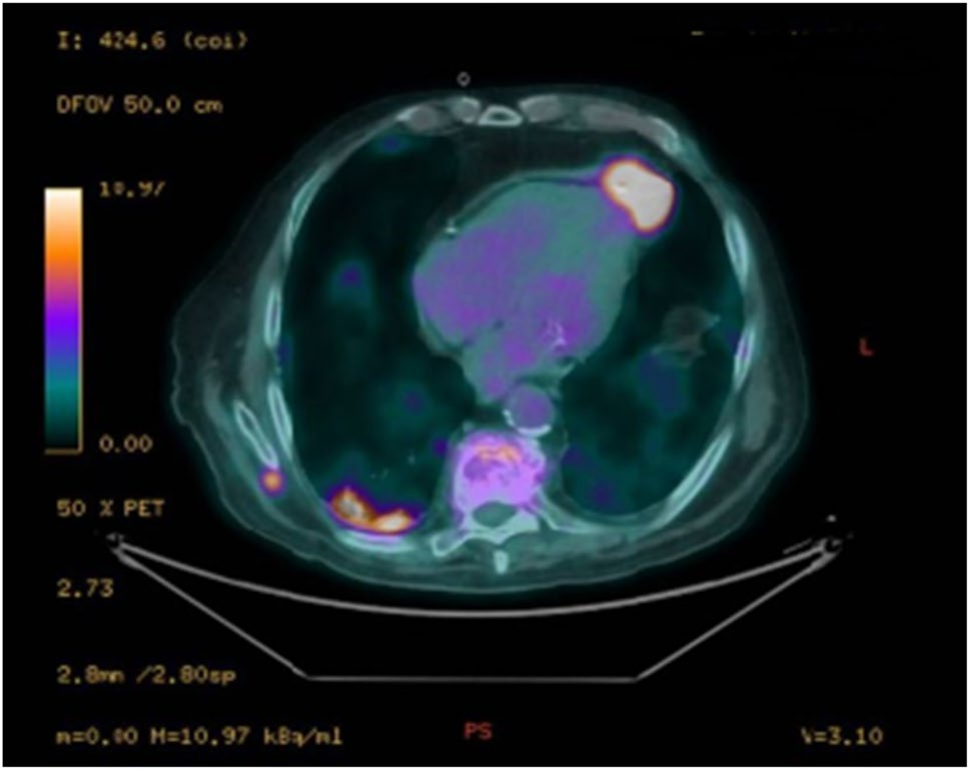
Images with hybrid technique with positron emission tomography-computed tomography (digital PET-CT) with 18F-FDG (219 MBq e.v.). The technique shows the presence of the collection localized near the cardiac apex, characterized by ring-shaped uptake (SUVmax 15.7)

## Data Availability

All data is in public domain.
